# Dermatofibrosarcoma Protuberans Over Right Lumbar Region: A Case Report

**DOI:** 10.7759/cureus.33208

**Published:** 2023-01-01

**Authors:** Bhavinee Pathak, Sabiha Maimoon

**Affiliations:** 1 Pathology, Narendra Kumar Prasadrao (NKP) Salve Institute of Medical Sciences, Nagpur, IND

**Keywords:** spindle cells, histopathology, case report, lumbar region, dermatofibrosarcoma protuberans

## Abstract

Dermatofibrosarcoma protuberans is a rare, low-grade dermal soft-tissue tumor with a high propensity for recurrence but a low propensity for metastatic spread. It mostly affects the head, neck, proximal extremities, and trunk. We report a case of dermatofibrosarcoma protuberans over the right lumbar region. The patient presented with swelling in the right lumbar region measuring 3 cm × 3 cm. The local region ultrasonography (USG) revealed a well-defined hypoechoic lesion in the subcutaneous area. The patient was provisionally diagnosed with a peripheral nerve sheath tumor under evaluation. USG-guided fine needle aspiration cytology* *(FNAC) suggested a spindle cell tumor. Wide local excision of the tumor was performed. Monomorphic spindle cells in a storiform pattern were observed by histological evaluation. The neighboring adipose tissue was invaded by the tumor cells in a honeycomb-shaped pattern. The histological features were suggestive of Dermatofibrosarcoma Protuberans. Due to the high likelihood of recurrence, it is crucial to monitor these patients for an extended period of time.

## Introduction

Darier and Ferrand first described dermatofibrosarcoma protuberans (DFSP) in 1924, while Hoffman first defined the term in 1925. It is an uncommon, soft tissue sarcoma (low to intermediate grade) that develops in the skin's dermal layer. Lesions may begin as a painless, skin-colored plaque with a potential dark red or blue staining and have a tendency to expand slowly. Later stages of DFSPs might enlarge and develop into protuberant or ulcerative lesions. They typically exhibit localized aggression and have the propensity to invade nearby structures, including the subcutaneous tissue, muscles, tendons, and even bone structures. However, metastatic disease is rarely documented [1]. According to reports, the incidence rate is 5/1,000,000 every year. The sex distribution is roughly equal, with a small female predominance, according to a previous study [2]. Most frequently, it affects the trunk, next to proximal extremities, and head and neck [3].

## Case presentation

Patient information and clinical findings

A 32-year-old female came with a chief complaint of swelling over the right side of the back since last year. It was gradual in onset, and there was a progressive increase in the size of the swelling. The patient complained of pain over the swelling. She had a history of cyst excision done four years back from the same site.

**Figure 1:**
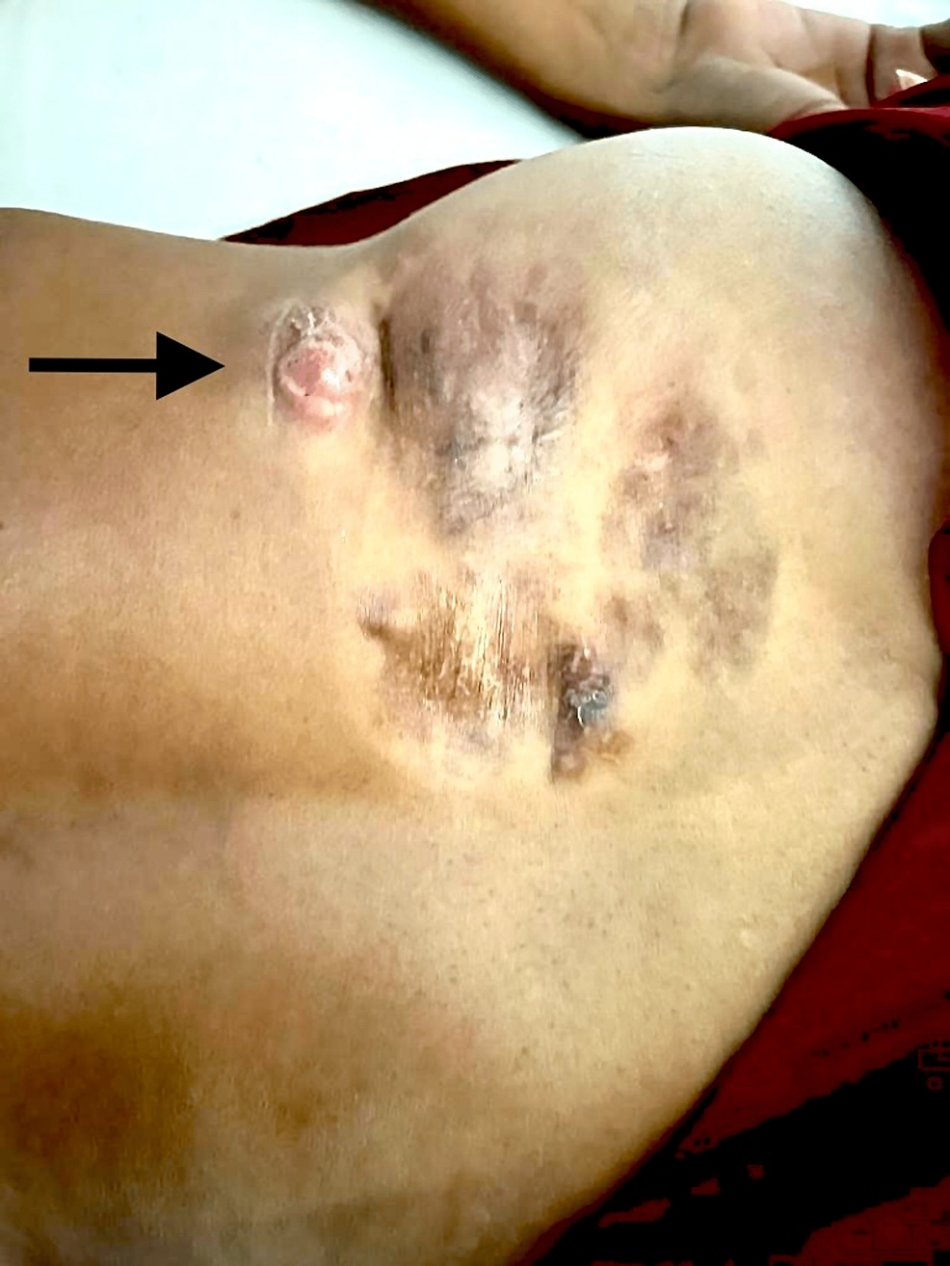
Clinical image of the lesion showing swelling

On local examination, a palpable lump of size 3 cm × 3 cm was present over the right side of the back (Figure 1). The swelling was firm, non-tender, and non-mobile. There were no areas of redness over the swelling. There was no local increase in temperature or any skin discoloration around the swelling. Systemic examination revealed no abnormalities.

Timeline of the current episode

The patient had swelling on the right side of the back for one year. Swelling gradually increased in size, involved the skin over the back, and caused pain and discomfort to the patient during the previous week. The patient had a similar complaint of cystic swelling over the same site four years back, which she had got excised. The patient exhibited generalized weakness. There was no history of discharge from the swelling. The patient does not have diabetes mellitus, asthma, tuberculosis (TB), hypertension, or any other known diseases.

Diagnostic assessment

The USG local region revealed a well-defined hypo-echoic lesion of size 3.8 cm x 2.1 cm x 3.5 cm in the subcutaneous area in the right lumbar region. It showed posterior acoustic enhancement and multiple septa within. Color Doppler imaging revealed a significant vascularity. The patient was provisionally diagnosed with peripheral nerve sheath tumor. USG-guided fine needle aspiration cytology (FNAC) was performed. 

Operative procedure

**Figure 2:**
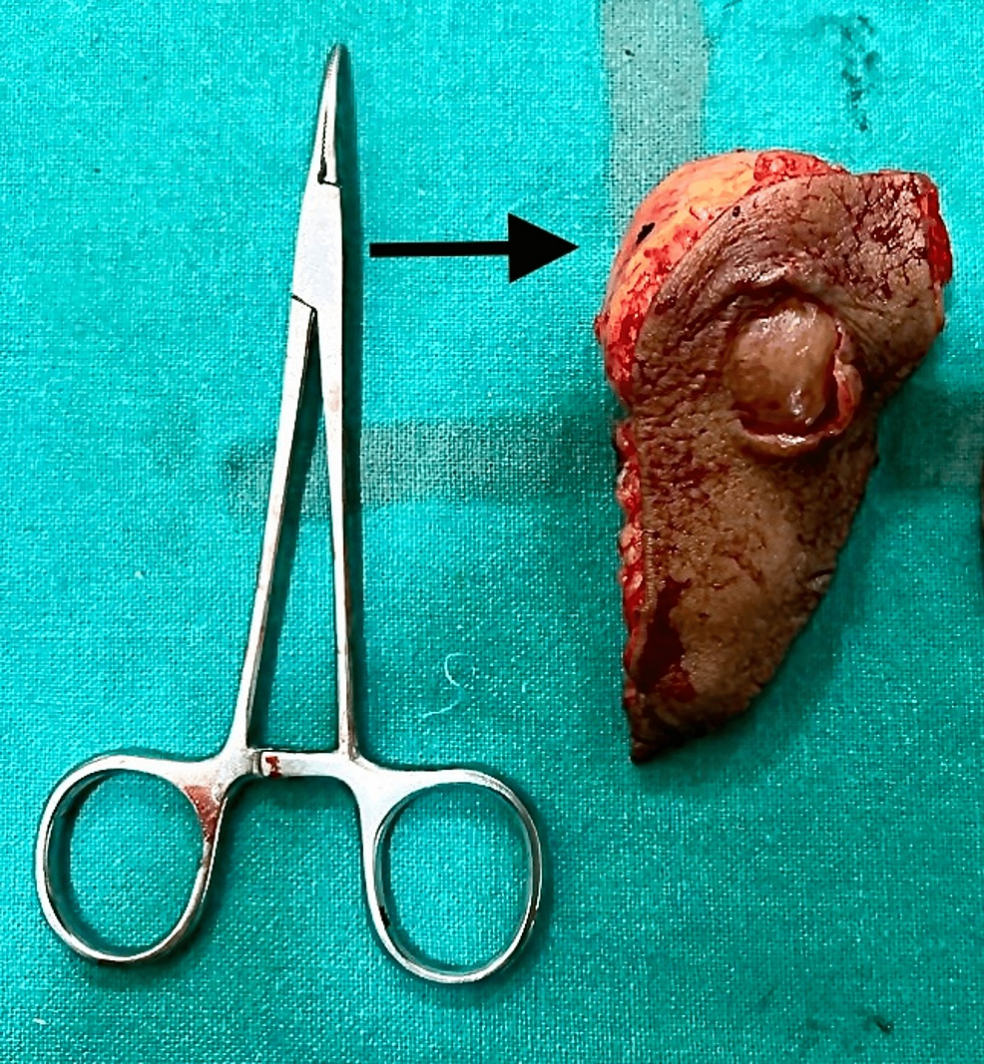
The excised tumor with skin

Wide local excision of the tumor was performed under general anesthesia, and the specimen (Figure 2) was sent for histological examination.

Diagnosis

**Figure 3:**
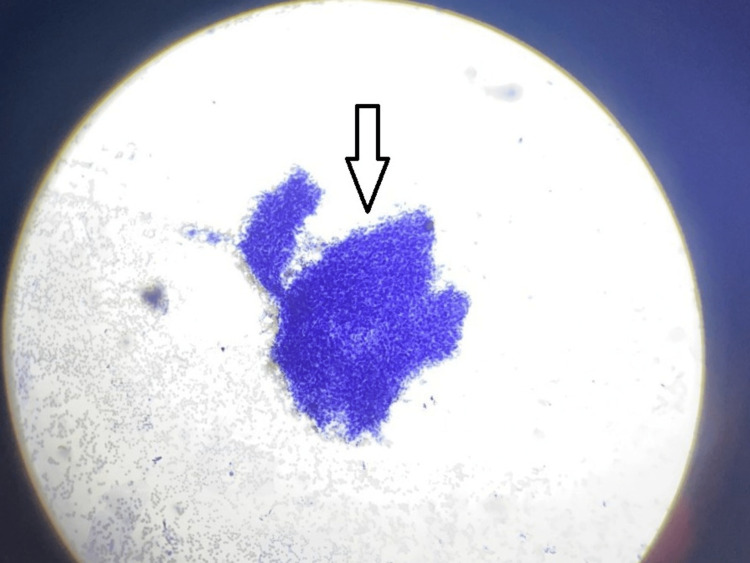
Haematoxylin & Eosin stain; 4x view fine needle aspirate with moderate cellularity

**Figure 4:**
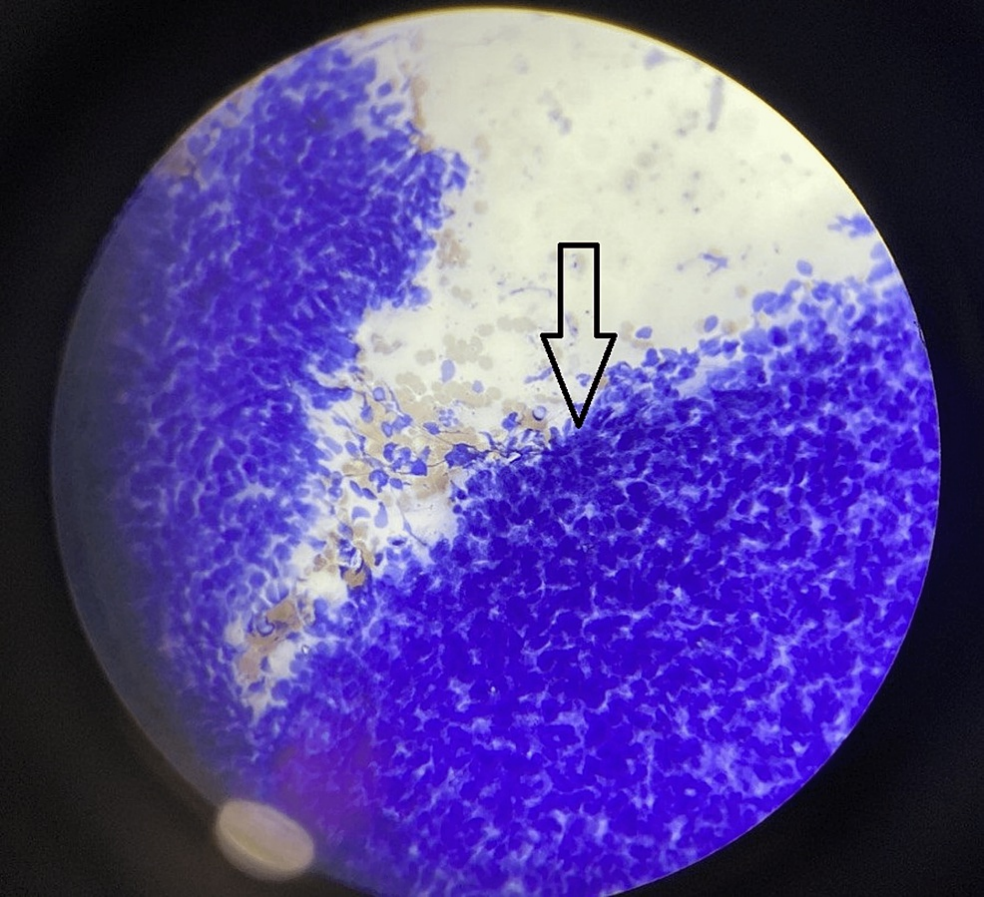
Haematoxylin & Eosin stain; 10x view showing tumor cells

**Figure 5:**
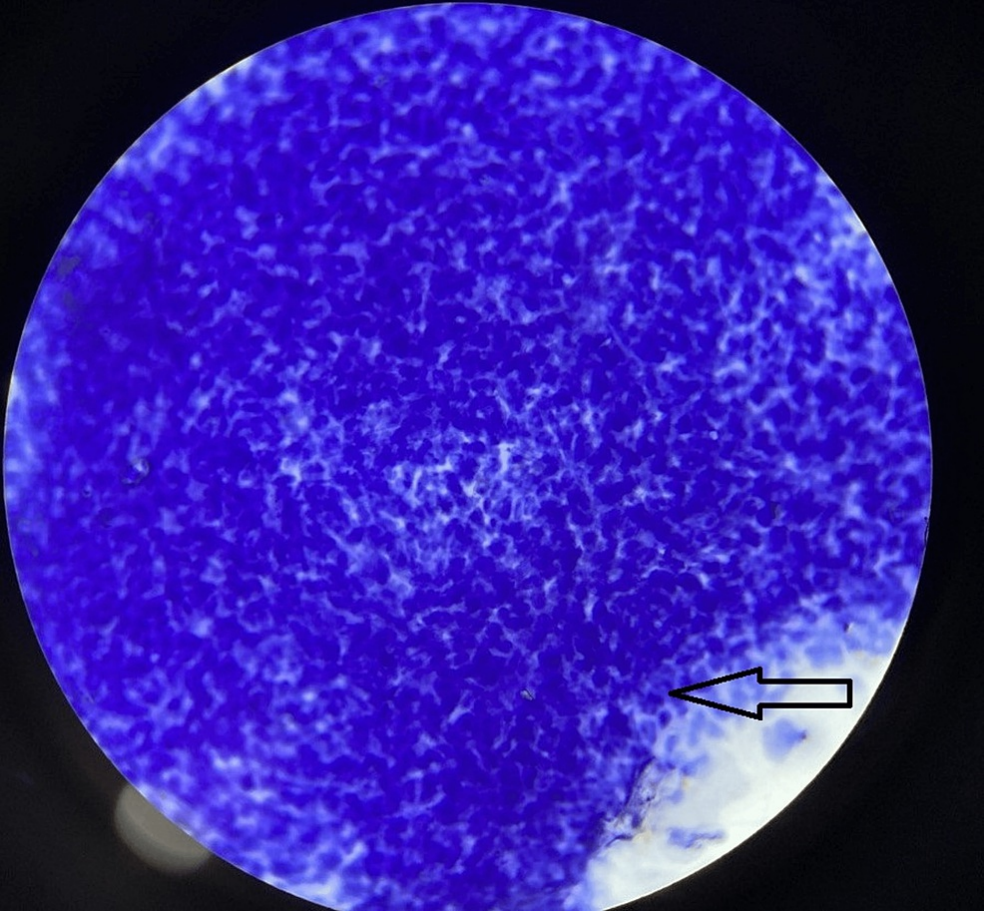
Haematoxylin & Eosin stain; 40x view showing plump and slender spindle cells

USG-guided fine-needle aspiration (FNA) smears revealed moderate cellularity (Figure 3), showing a cluster and dissociated singly scattered cells with ovoid nuclei and ill-defined cytoplasmic borders (Figure 4). A few plump and slender spindle cells were observed (Figure 5). An impression of cytological features in favor of a spindle cell tumor was made.

**Figure 6:**
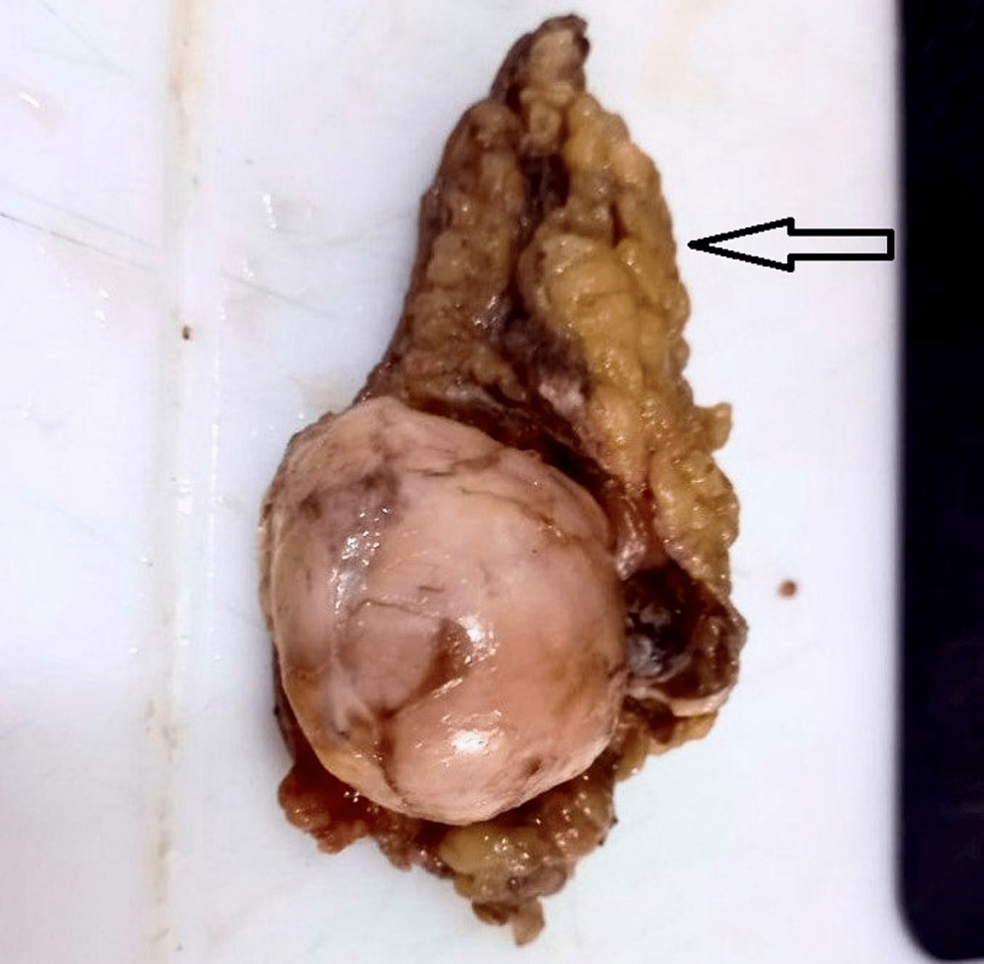
Excised tumor with fibroadipose tissue

**Figure 7:**
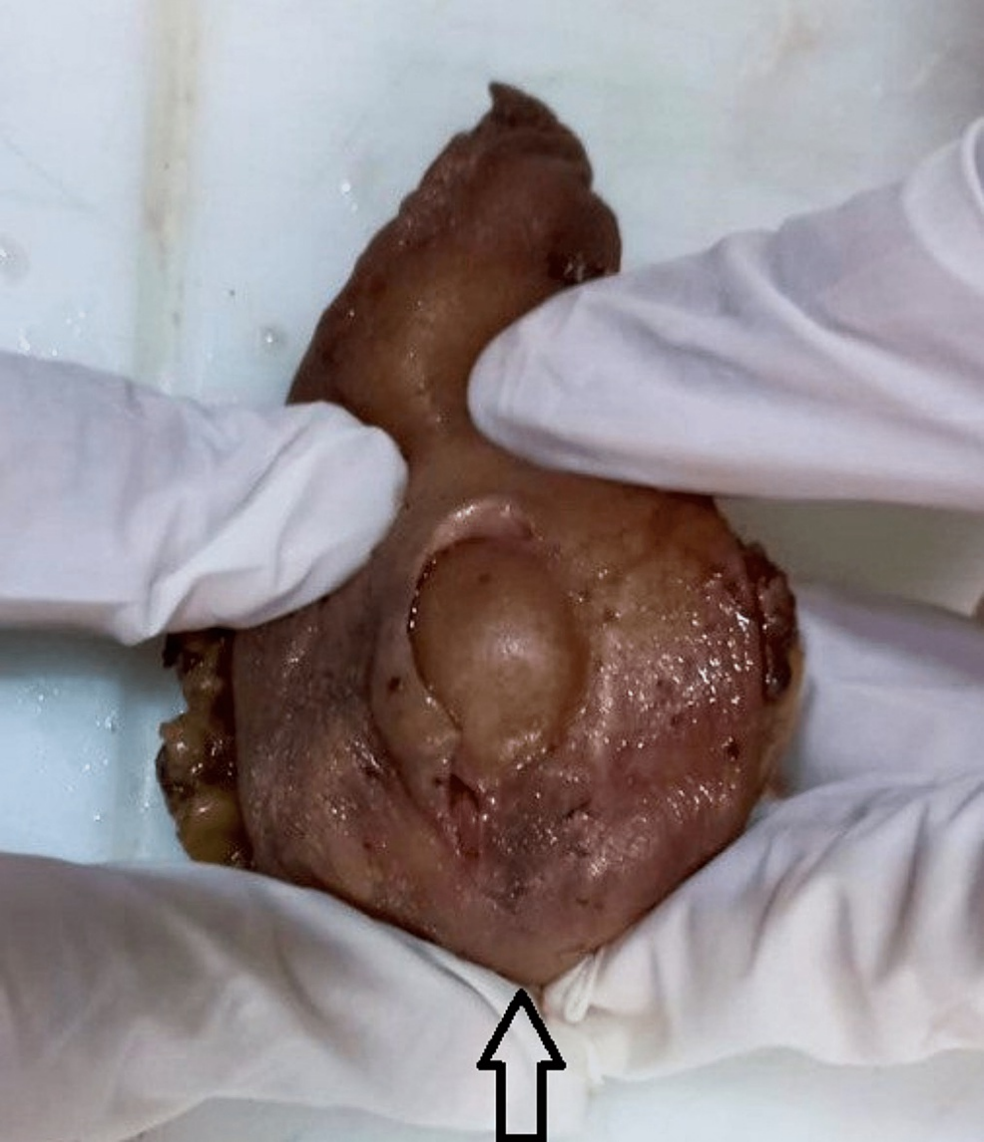
Tumor with attached skin

The excised tumor was subjected to a histological examination. It was a single globular specimen with attached fibroadipose tissues and skin (Figures 6, 7). The total dimensions of the specimen were 8 cm x 3.5 cm x 2.5 cm. The tumor was globular and white in color. The cut surface was homogenous white, soft to firm, and measured 3.5 cm x 3.5 cm x 3.5 cm in size.

**Figure 8:**
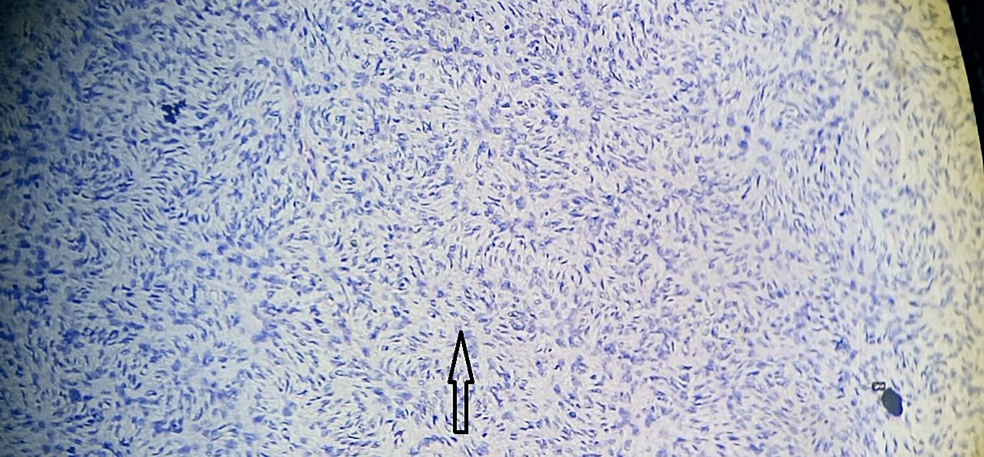
Haematoxylin & Eosin stain; 10x power view showing uniform, medium-sized spindle cells with a storiform pattern

**Figure 9:**
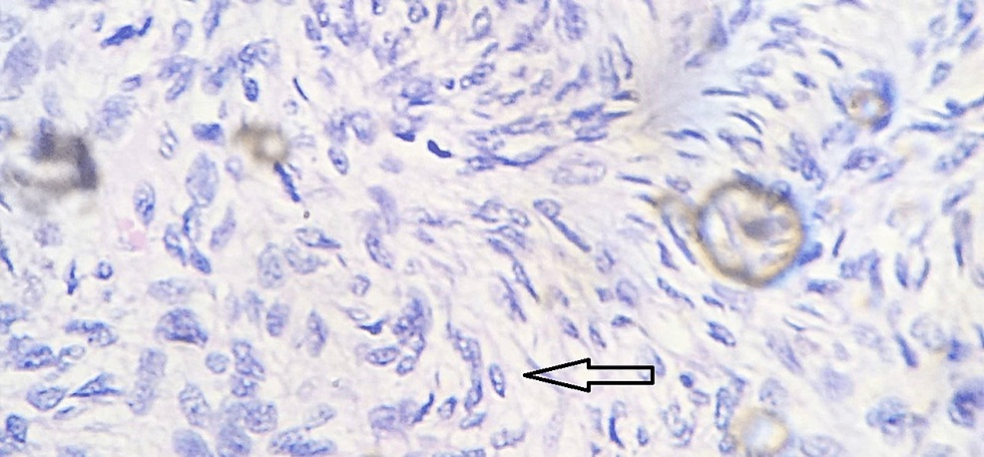
Haematoxylin & Eosin stain; 40x power view showing uniform, medium-sized spindle cells with a storiform or cartwheel pattern of growth

**Figure 10:**
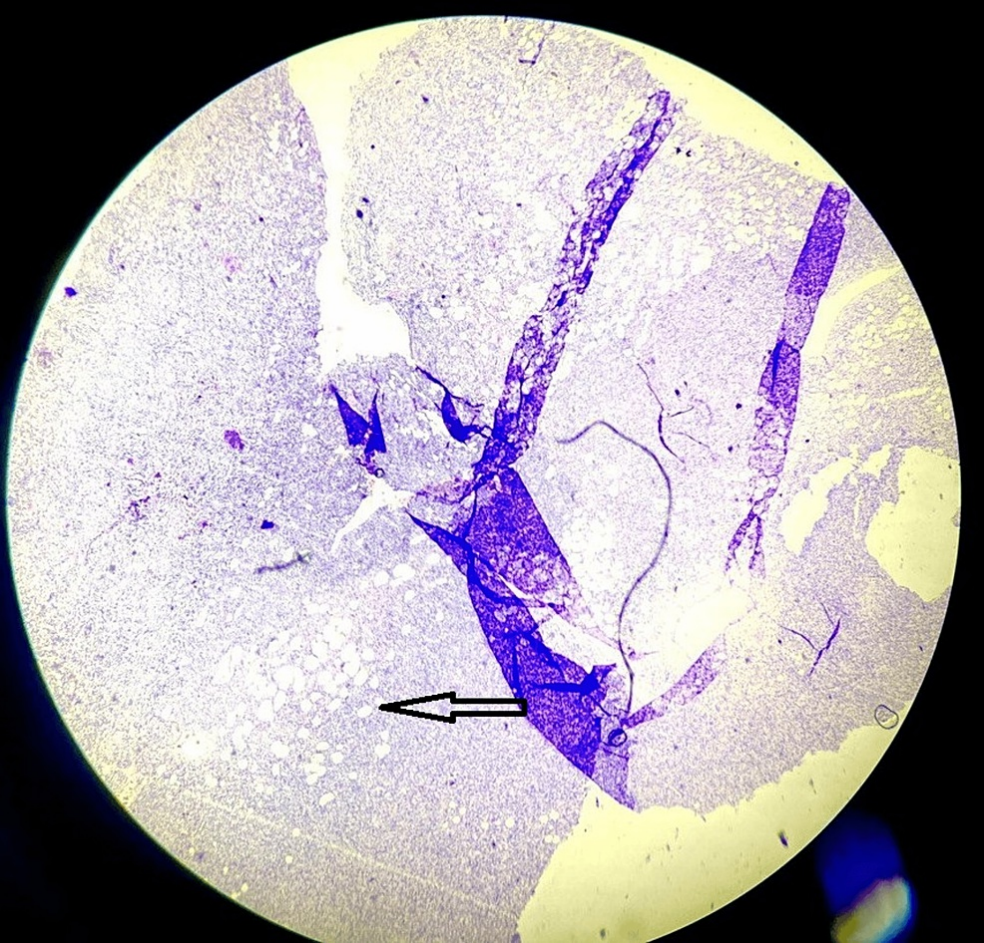
Haematoxylin & Eosin stain; 4x power view showing tumor cells extending in adipose tissue

**Figure 11:**
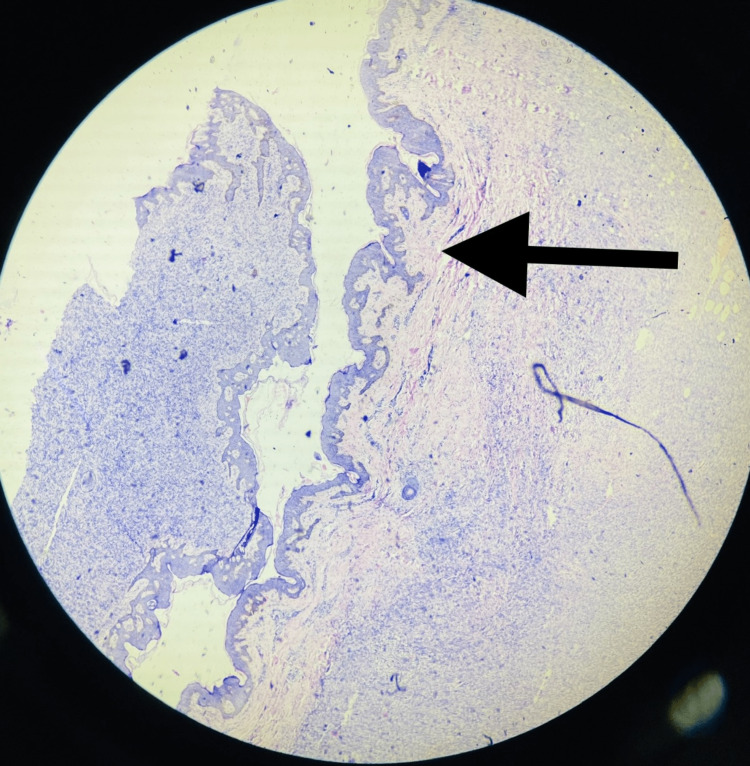
Haematoxylin & Eosin stain; 4x power view showing epidermis

On microscopy, sections from different areas revealed a nodular mass composed of a proliferation of mostly uniform, medium-sized spindle cells with a storiform or cartwheel pattern of growth (Figures 8, 9) placed in the dermis and extending into the subcutaneous adipose tissue (Figure 10). It was separated from the epidermis by a grenz zone. Adnexal structures were also embedded in the tumor. The overlying epidermis appeared normal (Figure 11). The tumor infiltrated the subcutaneous adipose tissue in a honeycomb pattern. Histological features suggestive of dermatofibrosarcoma protuberans were observed.

Follow-up and outcomes

Post-operation, the patient was relieved of acute symptoms of pain and swelling over the right side of her back. Her discomfort had subsided. Overall, the patient was doing quite well after the surgical intervention. 

## Discussion

DFSPs, a rare, slowly-growing malignant fibroblastic mesenchymal skin tumor, make up less than 0.1 percent of all malignant tumors and one percent of all soft tissue sarcomas [4]. The classic protuberant look of dermatofibrosarcoma is the clinical situation that is most frequently observed. Atrophic plaque-like lesions can occasionally be misdiagnosed as amorphea-like lesions [5-6]. The differential diagnosis of DFSP in its early stages includes lipomas, epidermal cysts, keloids, dermatofibromas & nodular fasciitis. The differential diagnosis in the later stages includes Kaposi sarcoma, pyogenic granuloma, and other soft tissue sarcomas [7].

DFSPs have been discovered to be predominantly hypo-echoic or mixed hyper-echoic on ultrasound, with mainly well-defined boundaries or irregular, with projections resembling pseudopodia [8]. DFSP's vascularity, a sign of malignancy, differs as well [9-10]. A difference isn't always achievable because lipomas can exhibit the same symptoms [11]. Furthermore, MRI investigations also do not help much in distinguishing dermatofibrosarcoma protuberans tumors from other soft tissue sarcomas, making them less precise [12]. The only reliable diagnostic technique is a histology study.

Diffuse invasion of the dermis and subcutis under a microscope identifies DFSP while typically sparing the epidermis and appendages of the skin. While entering fat lobules, it develops along preexisting fibrous septa, forming the characteristic honeycomb-shaped pattern. Dermatofibrosarcoma protuberans might sporadically manifest as an infiltrative subcutaneous tumor. Mitoses are uncommon, and atypia is modest. Tumor cells have immunohistochemical staining for CD34, vimentin, nestin, apolipoprotein D, and epithelial membrane antigen (EMA) [13].

Broad local excision is the preferred course of treatment with negative margins of 3-5 cm from the tumor edge. To obtain negative resection margins in cases when there is a chance that the bone will be involved, the periosteum or even a small amount of the bone may need to be removed. Depending on the resection margins, the rate of recurrence will change. Recurrence rates were under 5% in series that employed five-cm resection margins [1].

## Conclusions

DFSP is a rare tumor that grows slowly, with a female preponderance. It usually occurs around the age of 40 years. The storiform pattern of spindle cells is observed histologically. Treatment consists of complete surgical removal. There are many recurrences. The majority of local recurrences manifest during the first three years following surgery. Recurrences later than five years have also been documented, though. Therefore, it's crucial to keep an eye on these individuals over the long term.
